# Study protocol: a non-randomised community trial to evaluate the effectiveness of the communities that care prevention system in Germany

**DOI:** 10.1186/s12889-021-11935-x

**Published:** 2021-10-23

**Authors:** Dominik Röding, Renate Soellner, Maren Reder, Vera Birgel, Constantin Kleiner, Maike Stolz, Frederick Groeger-Roth, Christian Krauth, Ulla Walter

**Affiliations:** 1 Hannover Medical School, Institute for Epidemiology, Social Medicine and Health Systems Research, Hannover, Germany; 2 University of Hildesheim, Institute for Psychology, Hildesheim, Germany; 3 Crime Prevention Council of Lower Saxony, Hannover, Germany

**Keywords:** Effectiveness, Non-randomised, Quasi-experimental, Community trial, Youth, Problem behaviour, Capacity building, Intersectoral collaboration, Risk factors, Protective factors

## Abstract

**Background:**

The Communities That Care (CTC) prevention planning and implementation system trains communities throughout a five-phase cycle to (1) build capacity for prevention, (2) adopt science-based prevention, (3) assess the prevention needs of adolescents living in the community, (4) select, and (5) implement evidence-based programs according to their needs. After CTC proved to be effective and cost-effective in the U.S., it is being used by an increasing number of communities in Germany. The aim of this study is to evaluate the effectiveness and cost-effectiveness of CTC in Germany.

**Methods:**

Communities in CTC-phases 1 to 3 (*n* = 21) and individually-matched comparison communities (*n* = 21) were recruited for a non-randomised trial. To assess long-term outcomes, (1) a cohort of 5th Grade students will be surveyed biennially concerning behaviours (antisocial behaviour and substance use) and well-being as well as risk and protective factors. Additionally, (2) biennial cross-sectional surveys will be conducted in 6th, 8th, 10th, and 11th Grade in each community. To assess short-term outcomes, a cohort of ten key informants per community will be surveyed biennially concerning adoption of science-based prevention, collaboration, community support and community norms. (4) In a cross-sectional design, all ongoing prevention programs and activities in the communities will be assessed biennially and data will be collected about costs, implementation and other characteristics of the programs and activities. (5) To monitor the CTC implementation, the members of the local CTC-boards will be surveyed annually (cross-sectional design) about team functioning and coalition capacity. Data analysis will include general and generalised mixed models to assess the average treatment effect of CTC. Mediation analyses will be performed to test the logical model, e.g., adoption of science-based prevention as a mediator for the effectiveness of the CTC approach.

**Discussion:**

This is the first controlled study to evaluate the effectiveness of a comprehensive community prevention approach in Germany. Evaluating the effectiveness of CTC in Germany is an important prerequisite for further diffusion of the CTC approach.

**Trial registration:**

This study was registered with German Clinical Trial Register: DRKS00022819 on Aug 18, 2021.

## Background

Children and adolescent problem behaviour, such as antisocial behaviour, alcohol use, illicit drug use and delinquency, place a burden on healthy development and well-being [1–3]. Preventive programs aim to minimize these risk factors, strengthen corresponding protective factors, and promote resilience, which will be developed in a process of interaction between the individual and the environment [4–8]. In this context communities take a central function [9, 10]. They have the opportunity to shape this in a targeted manner via various institutions and settings as well as the establishment of intersectoral collaborations. But communities often have a lack of knowledge regarding prevention and health promotion, so that in many cases prevention programs and strategies are selected and implemented that show no or only limited effectiveness [11, 12] or have not yet been evaluated. Lack of program fidelity is also a problem [13, 14].

Building community networks for prevention strengthens the implementation and coordination of prevention programs [15–17]. So far, there are only a few studies that have investigated their effect on the health of the target group and can show effects [18–20]. A program used worldwide to build such community networks for prevention is Communities That Care (CTC).

CTC was developed in the U.S. in the 1980s. It supports communities in establishing healthy environments that enable children and adolescents to grow up safely and healthy. The Community Youth Development Study (CYDS) showed that CTC increased the adoption of a science-based approach to prevention, sectorial collaboration for prevention, and community support for prevention [21] as well as the number of implemented evidence-based prevention programs and the number of program participants in the community [22]. This resulted in lower incidences in smoking cigarettes, smokeless tobacco use, alcohol use and delinquent behaviour (Odds Ratios 1.41 to 2.34) compared to control communities [23]. The effects of CTC on youth problem behaviours were fully mediated by community adoption of a science-based approach to prevention [24]. Economic evaluations indicated that CTC may produce a return on investment of approximately US$10.23 for every dollar spent [25].

**Fig. 1:**
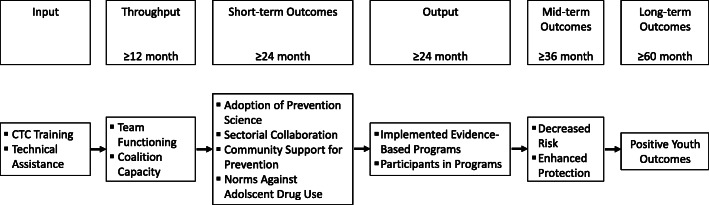
Communities That Care model of community change [cf. 6]

According to the CTC-model of community change it takes 1 year to observe improvements in throughputs, 2 years to observe effects in short-term outcomes and outputs, 3 years to observe mid-term outcomes and 5 years to observe long-term outcomes (Fig. 1). CTC’s theory of change and results from Brown et al. [24] suggest that the community adoption of a science-based approach to prevention is the primary mechanism of CTC.

In 2009, CTC was transferred to Germany and has since been implemented in 33 communities. In this context, the database “Grüne Liste Prävention” (www.gruene-liste-praevention.de) has been developed, which includes German-language evidence-based prevention programs. A feasibility study in Lower Saxony showed the transferability (i.e., high level of acceptance and identification with the CTC approach in the model communities) of CTC to the German context [26, 27], but an evaluation of its effectiveness has not been conducted in Germany to date. The aim of this study is to evaluate the effectiveness and cost-effectiveness of CTC in Germany.

## Methods and design

### Intervention

#### Intervention condition

**Fig. 2:**
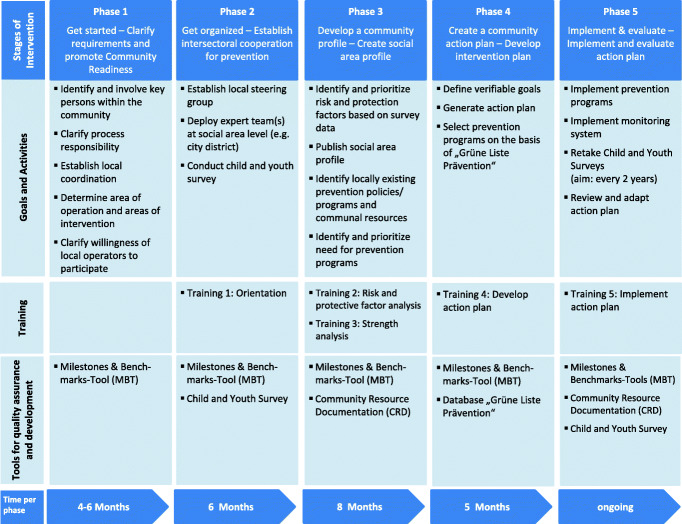
Overview of phases and components of the intervention [cf. 6]

CTC implementation is organised into five phases [28]. Training in the various phases of the CTC process is provided by the CTC Training and Support Center (German Prevention Congress) to encourage communities to achieve milestones and benchmarks. Specially developed instruments support quality assurance and development. Figure 2 shows the objectives, activities, training and instruments for quality assurance in the respective phases.

#### Comparison condition

For the comparison communities, it was not examined whether they use an elaborated system of community prevention. Due to the international push of intersectoral community-based prevention approaches [29] through the WHO Ottawa Charter on Health Promotion [30] it can be assumed that the majority of these communities work with a local intersectoral network for prevention and health promotion. Böhm and Gehne [31] give an overview of such networks in Germany.

### Design

**Fig. 3:**
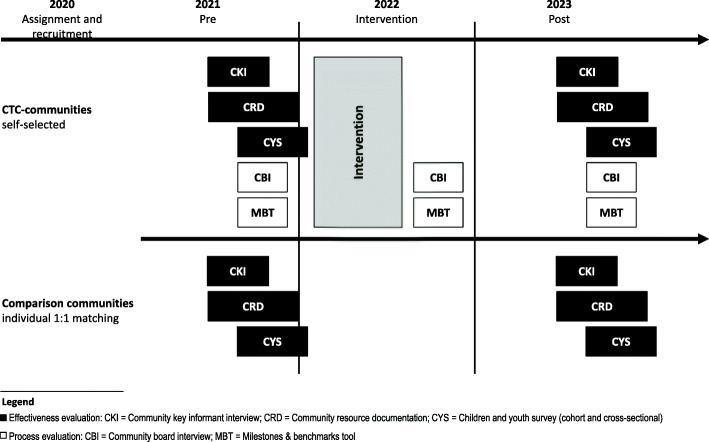
CTC-EFF Study design and measures

A quasi-experimental study will be conducted in small towns, rural communities and districts of major cities in four German federal states (Baden-Württemberg, Bavaria, Lower Saxony, Rhineland-Palatinate) to evaluate the effectiveness of CTC in Germany; hereinafter referred to as communities. The study is designed as a cluster non-randomised controlled trial [32]. To evaluate the effectiveness of CTC, data will be collected in a trend (repeated cross-sectional) and cohort design (Fig. 3). Additionally, the implementation of CTC will be evaluated (process evaluation). Funding provided, a follow-up will be conducted in 2025 and further years.

### Assignment method and eligibility

The unit of assignment is the community, the assignment method is self-selection. Individual 1:1-matching (see below) was employed to help minimize potential bias induced due to non-randomization.

Sampling was initiated in April 2020 by inviting all German CTC-communities that would be in Phases 1 to 3 in the CTC cycle by January 2021. To be included in the trial, communities had to have at least one secondary school and willingness to sign a cooperative agreement for study participation with the principal investigator Hannover Medical School. This resulted in a final sample of 21 CTC-communities (16 small towns and five city districts) by July 2021.

Immediately after a CTC-community was recruited, individual 1:1-matching was used to identify comparable communities in the same federal state. For this, a nearest-neighbor matching procedure was applied to community-level data from official statistics 2017 (www.inkar.de) and police crime statistics (PCS) 2019, using indicators of municipality type, community development (growth vs. stable vs. shrinkage), long-term unemployment, fiscal power, criminality, vandalism, drug use as well as shoplifting rate in youth. For each recruited CTC-community, the comparison community with the best-matching score was requested to participate in the study. In case the community was not interested in participating, the next most similar community was contacted. Comparison communities were only eligible to participate in the study if (1) they had at least one secondary school, (2) they were not located in a county that implements CTC, (3) they were not directly adjacent to a CTC-community, and (5) they signed a cooperative agreement for study participation with the principal investigator Hannover Medical School. Overall, a sample of 21 matched pairs was recruited.

### Participants and eligibility

#### Community key informants

In each study community, people in leadership positions (e.g., mayors, city managers, police chiefs, school superintendents, business leaders, heads of social service agencies) will be invited to participate. These leaderships will be identified via the research staff and by using a snowball sampling method. Only those persons who have consented to the data collection will be interviewed.

#### Prevention actors

Prevention actors (directors of community agencies and coalitions, prevention program coordinators and staff, school principals, teachers) who are responsible for prevention activities and programs in the study communities will be invited to participate. These actors will be identified via the research staff and by using a snowball sampling method. Only those persons who have consented to the data collection will be interviewed.

#### Community board members

All members of the local CTC-prevention networks (community boards) will be invited to participate. Only those persons who have consented to the data collection will be interviewed.

#### CTC-trainers and local CTC-coordinators

CTC trainers and local CTC coordinators are invited to participate and to consent to the data collection.

#### Children and youth

Every secondary school in the study communities and relevant schools in the vicinity of these communities attended by a substantial number of community adolescents will be invited to participate. The survey will depened on the support of (1) school principals and (2) class teachers. Additionally, written informed consent of the (3) parents will be mandatory. Finally, (5) each student will have to consent to the survey. For the cohort survey, students of Grade 5 will be invited to participate. For the cross-sectional survey, students of Grades 6, 8, 10, 11 of secondary schools as well as students aged < 18 years of vocational schools are eligible.

### Measures

#### Effectiveness evaluation measures

##### Community key informant interviews (CKI)

A translated and adapted version of the CKI which was developed by Arthur, Glaser and Hawkins [33] will be conducted using computer-assisted telephone interviews. Data will be collected concerning (1) major changes or events in the community that could affect the local prevention activities; (2) the adoption of a science-based approach to prevention; (3) intersectoral collaboration for prevention; (5) community support for prevention; and (5) community norms against adolescent drug use.

##### Community resource documentation (CRD)

A translated and modified version of the instrument set [8, 22] will be used to measure the type, number, and scope of prevention activities. Using computer-assisted telephone interviews, data will be collected concerning the names of all evidence-based programs that were conducted in the community, how many participants each program has reached and the fidelity of this program. Program fidelity will include the subcategories program adaptation, adherence, dosage, participant responsiveness and oversight and will be based on previous items measuring program fidelity within CTC-contexts (see [22, 34, 35]). Additionally, cost data of the prevention activities and programs will be collected.

##### Children and youth survey (CYS)

A translated and adapted version of the US CTC survey [33], which was applied in Lower Saxony-wide surveys to establish reference values [36, 37], will be used to collect data on (1) socio-demographic variables (gender, age, parents living together, family size, language background, socio-economic status); (2) self-reported problem areas (violence, delinquency, substance use, truancy/temporary school drop-out, depressive symptoms, mobbing, intimate partner violence, discriminatory behavior, life and health satisfaction); (3) risk factors (community, family, school, peer/individual); (5) protective factors (community, family, school, peer/individual); (5) COVID-19 questions based on the JuCo study [38]. Each survey will take about 45 min and will take place during a school lesson as an online survey. For schools unable to provide an internet connection or computers, a paper version of the questionnaire will be available.

#### Process evaluation measures

##### Community board interview (CBI)

A translated and adapted version of the CBI will be used to monitor the implementation of CTC in the intervention communities. The CBI captures constructs associated with developing and maintaining an effective community coalition [39, 40]. The construct community coalition capacity captures five components [39]: (1) members’ substantive knowledge of prevention; (2) members’ acquisition of new skills; (3) members’ attitudes toward CTC; (4) organizational linkages to the coalition across community sectors; (5) and members’ perceptions of their coalition’s influence on organizations in the community on the development of intersectoral cooperation. The construct team functioning includes four components [40]: (1) goal directedness; (2) efficiency; (3) opportunities for participation; and (4) cohesion.

##### Milestones and benchmarks tool (MBT)

Additional measures of CTC implementation are obtained from ratings of the CTC Milestones and Benchmarks (see [41]). The CTC training materials describe “milestones” and “benchmarks” that are to be achieved during the five phases of CTC system implementation. The milestones are goals to be met by communities, and the benchmarks are the actions that community members take or conditions that must be present to achieve those goals. The local CTC-coordinators and the CTC-trainers are asked to send their ratings annually to the research team of this study.

### Outcomes

#### Primary outcomes

##### Primary short-term outcome

Based on the CYDS [21], we hypothesize that, compared to comparison communities, CTC-communities will (1) have stronger improvements regarding the adoption of a science-based approach to preventing youth problem behaviour, (2) have stronger improvements in intersectoral collaboration for prevention, and (3) have stronger improvements in community support for prevention.

##### Primary long-term outcomes

We hypothesize that CTC-communities will show lower prevalence and incidence of (1) antisocial behaviour and (2) substance use as well as (3) higher levels of well-being.

#### Secondary outcomes

##### Secondary short-term outcomes

Based on the CYDS [34], we hypothesize that, compared to comparison communities, CTC-communities will (1) implement more evidence-based prevention programs, (2) reach more persons with the implemented prevention programs, and (3) conduct the prevention programs with a higher fidelity.

##### Secondary long-term outcomes

We hypothesize that risk factors (community, family, school, peer/individual) which have been prioritized by the CTC-communities decrease whereas prioritized protective factors increase.

### Sample size calculation

For sample size calculations https://researchmethodsresources.nih.gov (Research Methods Resources: National Institutes of Health 2021) was used. Sample size calculations were conducted for the primary outcome in the short-term and long-term.

#### Primary short-term outcome: adoption score of science-based prevention

The calculation is for a net difference in a cohort study. We assume a type 1 error rate of 5%, desired power of 95%, correlation over time for the outcome variable of 0.6. A priori matching of the communities is considered with a correlation between matching factors and the outcome of 0.1 (individual level) and 0.2 (community level). Based on Brown et al. [21], regression adjustment for respondent characteristics is considered. An intraclass correlation coefficient (ICC) of 0.181 is assumed and for comparison communities a probability of 0.13 as well as for CTC-communities a probability of 0.34 of being in stage five of the adoption score (0–5) at post-test. Based on Muellmann et al. [42], 10 individuals (key informants) per cluster were considered. This results in a net sample size of 17 CTC-communities and 17 comparison communities with an average of 10 participants (key informants). Assuming a loss-to-follow-up of two matched pairs (four communities), a sample of 38 communities (19 matched pairs) and a total of 380 participants (key informants) is required.

#### Primary long-term outcomes: well-being, antisocial behavior, substance use

The sample sizes for the cohort and repeated cross-sectional design were calculated separately though largely relying on the same assumptions. Considering the heterogenous effects of the CYDS at the different follow-up intervals [43–45], we assume small effects (d = .2) for our primary outcomes in both the cohort and repeated cross-sectional design. We assumed a type 1 error rate of 5%, desired power of 80%, SD = 1, no adjustment for covariates, intraclass correlation of 0.013 [46]. An a priori matching of the communities (see below) was also considered with a correlation between matching factors and the outcome of 0.1 (individual level) and 0.3 (cluster level). For the cohort design, the calculation was based on a net difference assuming an average number of 150 individuals per cluster (community) and a correlation over time for the outcome variable of 0.7 (individual level) and 0.6 (cluster-level). For the cross-sectional design, the calculation was based on a simple difference assuming an average number of 450 individuals per cluster (community). Both calculations result in a required sample of 14 communities (7 per condition). The overall sample of students required for the cohort survey is *n* = 2100 and for the cross-sectional survey *n* = 6300. Assuming a loss-to-follow-up of two matched pairs (four communities), this results in a sample of 18 communities (9 matched pairs).

### Statistical methods

#### Effectiveness evaluation

##### Short-term outcomes

Changes in primary short-term outcomes (i.e., adoption of science-based prevention) will be assessed by using a three-level hierarchical linear model (HLM) [47] and a cumulative probability model [48] to model changes in each of the outcomes as a function of survey year (at Level 1) nested within respondents (at Level 2), in turn, nested within communities (at Level 3).

##### Long-term outcomes

Primary and secondary outcomes will be tested for differences between intervention and control by using either complex or multilevel structural equation models depending on the necessity to adjust for baseline differences between the student populations of the matched communities. Models will incorporate the nested data structure of students in classes/schools in communities in matched pairs. Additionally, baseline levels of the outcomes will be included in the cohort analysis. The primary analysis focuses on the effect of intervention group on the primary outcomes at the community level. As secondary analyses, hypothesized mediation and moderation paths and effects at school and individual level will be tested.

#### Economic evaluation

The primary short-term and long-term outcomes collected will serve as effect parameters for the cost-effectiveness analysis. Cost has two parts: the quantitative measurement of resource use and the assignment of unit costs or prices [49]. The resource quantities associated with the implementation of the CTC process and prevention programs will be divided into personnel and material resources, which will both be recorded using additional modules of the CBI and CRD instruments. The time and material expenditure determined will be assessed in monetary terms using wage and price documentation.

Costs will be calculated for (1) CTC communities (including costs for the CTC process and prevention programs) and for (2) comparison communities (including costs for prevention programs) in relation to CTC communities. All analyses are carried out on three levels: (a) average costs per community, (b) average and median costs per resident, (c) average and median costs per youth. Because the size of the communities can differ significantly whereas some cost parameters (e.g., CTC coordinators salaries, need for training in the community, costs of purchasing program curricula) arise regardless of the number of residents and young people within a community, average intervention costs might be skewed by a few communities at extremes of the population distribution [25]. Therefore, the median will be calculated in addition to the mean, since this is a more robust measure against outliers. A linear regression with community size as an independent variable will be another way to take differences in population distribution into account.

In a first step the overall costs for prevention programs at the beginning and at the end of the study period will be compared for both described variants. The same analyses will be done, differentiated in financial resources spent for evidence-based and non-evidence-based programs. In addition, we will determine the share that individual cost parameters have in the total costs for the CTC process.

In the cost-effectiveness analysis, the ascertained incremental costs for the CTC process and implementation of prevention programs will be compared with the incremental effectiveness of the CTC communities (compared to the comparison communities). The adoption of science-based prevention serves as the primary effect parameter in the short-term and positive youth outcomes (Fig. 1) will be evaluated as primary effect parameters in the long-term (funding provided). Calculation of confidence intervals and bootstrapping will be used to map the uncertainties in determining the costs and the incremental cost-effectiveness. If there is follow-up funding after 2023, it is planned to model the long-term effects (e.g., secondary diseases) and the long-term benefits (quality of life, life expectancy and monetary benefits) of the implementation of CTC.

## Discussion

This is the first community trial to evaluate the effectiveness of a comprehensive community prevention approach in Germany. We expect that CTC will be associated with significant effects on risk and protective factors (secondary long-term outcomes) as well as student behaviour and well-being (primary long-term outcomes) through the adoption of a science-based approach of community prevention (primary short-term outcome) and the number of implemented evidence-based prevention programs and reached persons. While the evaluation of short-term outcomes can be conducted during the currently funded study period (April 2020 to December 2023), the evaluation of long-term outcomes is dependent on the study receiving follow-up funding of at least 2 years (see Fig. 1).

## Data Availability

Data sharing is not applicable to this article as no datasets were generated or analyzed during the current study.

## Abbreviations

CBI: Community Board Members Interview
CKI: Community Key Informants Interview
COVID-19: Corona Virus Disease 2019
CRD: Community Resource Documentation
CTC: Communities That Care
CTC-EFF: Effectiveness of the community preventions system Communities That Care
CYDS: Community Youth Development Study
CYS: Children and Youth Survey
d: Cohen’s d
Fig.: Figure
MBT: Milestones and Benchmarks Tool
SD: Standard Deviation
U.S.: United States
WHO: World Health Organization
